# Effects of comorbid chronic kidney disease on mortality in idiopathic pulmonary fibrosis patients and influence of pirfenidone

**DOI:** 10.1038/s41598-023-46506-0

**Published:** 2023-11-07

**Authors:** Yong Suk Jo, Kyung Joo Kim, Chin Kook Rhee, Yong Hyun Kim

**Affiliations:** 1 Division of Pulmonary and Critical Care Medicine, Department of Internal Medicine, College of Medicine, Seoul St. Mary’s Hospital, The Catholic University of Korea, Seoul, Republic of Korea; 2 Division of Pulmonary, Allergy and Critical Care Medicine, Department of Internal Medicine, Bucheon St. Mary’s Hospital, College of Medicine, The Catholic University of Korea, Seoul, Republic of Korea

**Keywords:** Chronic kidney disease, Respiratory tract diseases

## Abstract

Chronic kidney disease (CKD) is a comorbidity in idiopathic pulmonary fibrosis (IPF), and managing IPF with CKD is challenging due to limited options for antifibrotic therapy. The aim of this study was to examine the prevalence of CKD and prescription status of pirfenidone in IPF patients and to analyze its impact on mortality. Data from the Korean National Health Insurance Service (NHIS) database between October 2015 and September 2021 were used. IPF and CKD were defined based on both International Classification of Diseases 10th Revision (ICD-10) codes and Rare Intractable Disease (RID) codes. The risk of mortality was assessed based on accompanying CKD with or without antifibrotic therapy. Among 5038 patients with IPF, 8.4% had comorbid CKD and 83.3% with CKD did not receive renal replacement therapy (RRT). Patients with IPF and CKD were older, predominantly male, and had more frequent comorbidities such as cardiovascular disease and diabetes mellitus than subjects without CKD. Pirfenidone was prescribed to 105 (24.6%) of 426 CKD patients, and 89.5% of them did not receive RRT. Pirfenidone was also prescribed to 775 (16.8%) of 4612 IPF patients without CKD. Significant difference was not found in all-cause mortality between the IPF patients with or without CKD regardless of pirfenidone treatment. The use of antifibrotics in IPF patients with CKD is limited due to CKD severity; however, evidence is lacking. Mortality did not increase with accompanying CKD regardless of antifibrotic use. Further research on IPF and CKD is needed.

## Introduction

Idiopathic pulmonary fibrosis (IPF) is the most common, progressive fibrosing interstitial lung disease (ILD) of unknown causes with poor prognosis. IPF usually develops in the sixth to eighth decades of life and has a high mortality rate, with approximately 50% of IPF patients dying within 2–3 years, which is worse than many malignancies^1,2^. However, the natural history of IPF is highly variable. In addition to the management of IPF, the effects of comorbidities on the prognosis of IPF should be considered. IPF has both pulmonary and extrapulmonary comorbidities. Lung cancer, pulmonary hypertension, gastroesophageal reflux disease, and cardiovascular disease have been reported as prevalent comorbidities of IPF and are associated with increased mortality in IPF patients^3–7^. Almost all IPF patients have at least one comorbidity, and more than one-third have more than four comorbidities^8^. The greater number of comorbidities present in IPF patients is associated with significantly reduced survival.

Chronic kidney disease (CKD) is a common disease with prevalence increasing with age and is estimated to affect 11–13% of the population^9^. Well-known factors associated with CKD include age, hypertension, diabetes mellitus, smoking history, and cardiovascular disease^10^. CKD is a leading cause of death worldwide, and patients with CKD at an advanced stage have a higher risk of mortality^11,12^. In CKD patients, the prevalence of both obstructive and restrictive pulmonary dysfunction has increased, and the more severe is the renal impairment, the greater is the risk of developing pulmonary disease^13^. CKD is also regarded a comorbid condition of IPF. Among 123 IPF patients, 37 (30%) were diagnosed with CKD and showed significantly worse survival compared with IPF patients without CKD^14^. In a Danish ILD cohort that included 150 IPF patients, almost all patients had at least one comorbidity, including CKD in 27 patients (18%)^15^.

Currently, a curative treatment for IPF does not exist, and treatment options include both non-pharmacological and pharmacological approaches to slow disease progression and alleviate symptoms. However, managing patients with IPF and CKD is challenging because information on the effectiveness and safety of antifibrotics in those patients is lacking due to the absence of specific clinical trials. Therefore, this study aimed to investigate the prevalence of CKD, prescription status of pirfenidone, and effects on mortality in patients with IPF with or without CKD.

## Methods

### Data source

We conducted a population-based retrospective cohort study using the National Health Insurance Service (NHIS) database, a single mandatory health coverage system in South Korea. This unique compulsory single-payer health insurance system covers approximately 97% of all Korean citizens. The Korean government-affiliated Health Insurance Review and Assessment (HIRA) collects all the claims from hospitals and evaluates adequacy of patient use of medical facilities. Thus, this database contains socio-demographic data on all subscribers including age, sex, and income as well as information on healthcare utilization for inpatients and outpatients, which allows the assessment of major and minor diagnoses using International Classification of Diseases 10th Revision (ICD-10) codes, as well as procedures and prescriptions data.

### Definition of patients with IPF

IPF was defined based on both ICD-10 codes for IPF (J841 or J8418) and the Rare Intractable Diseases (RID) code V236. Generally, the diagnosis and management of IPF require a multidisciplinary approach; therefore, we only included claims data from referral hospitals. Pirfenidone has been covered by health insurance since October 2015 in Republic of Korea; therefore, we analyzed prescription data on pirfenidone from October 2015 to September 2021. The codes for specific drugs identifiable in the HIRA database allow assessment of the association with pirfenidone and lung cancer in IPF patients.

**Figure 1:**
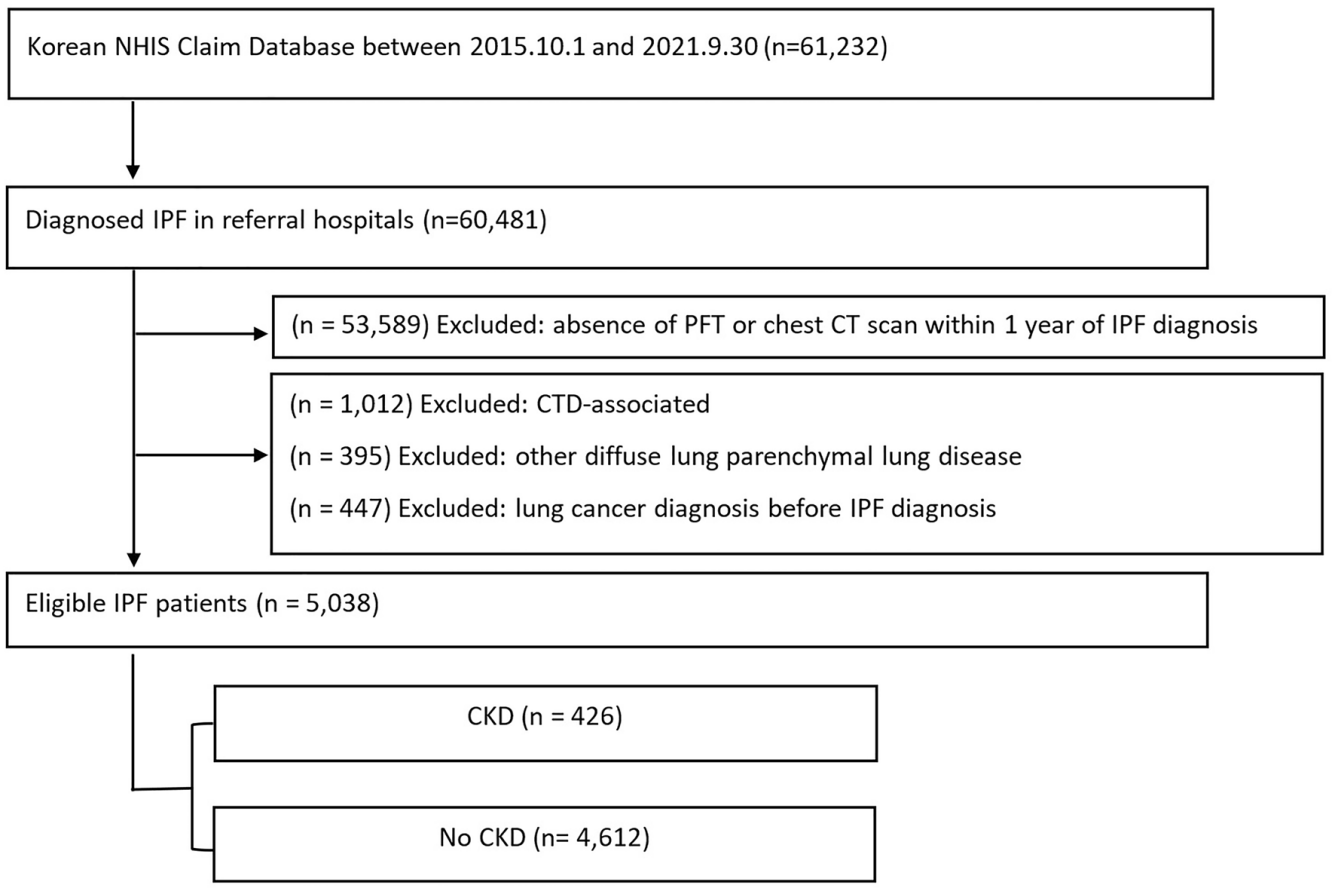
Flow chart of the study. CKD, chronic kidney disease; CT, computed tomography; CTD, connective tissue disease; IPF, idiopathic pulmonary fibrosis, NHIS, National Health Insurance System; PFT, pulmonary function test.

As shown in Fig. 1, 60,481 IPF patients were identified, and 53,589 subjects who did not undergo pulmonary function test and/or chest computed tomography (CT) within one year of IPF diagnosis were excluded. To clarify the definition of IPF, other forms of ILD were excluded (See Table S1).

### Definition of CKD

CKD was defined based on the ICD-10 code (N18), and we further specified patients with CKD by incorporating RID codes for hemodialysis (HD, V001), peritoneal dialysis (PD, V003), and kidney transplantation (KT, K005) and procedure codes for HD (O7020, O7021, O9991), PD (O2016, O707), and KT (R3280). CKD patients were divided into two groups: CKD patients on renal replacement therapy (RRT) including HD, PD, and KT and CKD patients not on RRT.

### Outcomes

The main purpose of this study was to assess prevalence of CKD; prescription rate of pirfenidone in patients with IPF and CKD; and risk of all-cause mortality based on comorbid CKD and pirfenidone treatment. Mortality cases were identified based on absence of any claim data from health care facilities for more than one year.

### Statistical analysis

Clinical features between groups divided based on comorbid CKD and pirfenidone therapy were compared and presented as number (%) and means with standard deviations. We used the Kaplan–Meier curve for comparison of cumulative all-cause mortality and analyzed mortality rate depending on comorbid CKD and/or pirfenidone treatment. The log-rank test was used to evaluate statistical difference among the groups. The effects of CKD and pirfenidone treatment on outcome were examined using Cox proportional hazards analysis.

All analyses were two-sided and conducted at a significance level of 0.05 unless otherwise noted. All analyses were performed using SAS software, version 9.2 (SAS Institute Inc., Cary, NC, USA).

### Ethics approval and consent to participate

The Institutional Review Board (IRB) of Seoul St Mary’s Hospital approved the study protocol (No. KC22ZASE0545) and has waived the need for informed consent due to the retrospective study. The study was performed in accordance with the principles of the Declaration of Helsinki concerning the ethical principles for medical research.

### Financial/nonfinancial disclosures

None of the authors have any financial relationships with a commercial entity with an interest in the subject of this manuscript.

## Results

### Baseline characteristics

Among the 5038 IPF patients, 426 (8.4%) had comorbid CKD (Fig. 1). Among the patients with both IPF and CKD, 71 (16.7%) were administered RRT: 61 were on HD, 5 were on PD, and 5 received a KT.

Characteristics of IPF patients with or without CKD are presented in Table S2. Compared with subjects without CKD, patients with CKD were older (mean age 76.8 years *vs*. 73.0 years), more likely to be male (83.3% *vs*. 71.4%), and more likely to have certain comorbidities such as cardiovascular disease including myocardial infarction, congestive heart failure, atrial fibrillation, hypertension, peripheral vascular disease, cerebrovascular accident, diabetes mellitus, and malignancy other than lung cancer.

Table 1 shows characteristics of IPF patients with or without CKD and comparison between the two groups based on whether they were treated with pirfenidone. Pirfenidone was administered to 24.6% of CKD patients and 16.8% of subjects without CKD. Patients taking pirfenidone were slightly younger in the CKD group and more likely to be male in both groups. Regarding comorbidities, significant differences were not observed in subjects with CKD who did or did not receive pirfenidone therapy except in patients with lung cancer. In the group without CKD, patients treated with pirfenidone were more likely to have certain comorbidities such as hypertension, diabetes mellitus, atrial fibrillation, and lung cancer.

**Table 1:** Clinical characteristics of IPF with or without CKD and use of pirfenidone.

Characteristics	IPF group					
	With CKD (n = 426)		P value	Without CKD (n = 4612)		P value
	Pirfenidone (−)	Pirfenidone (+)		Pirfenidone (−)	Pirfenidone (+)	
Number	321 (75.4)	105 (24.6)		3837 (83.2)	775 (16.8)	
Patients related						
Age, years	77.31 ± 8.99	75.11 ± 8.37	0.027	72.91 ± 9.94	73.26 ± 8.34	< 0.001
Sex, male (%)	261 (81.3)	94 (89.5)	0.049	2665 (69.5)	626 (80.8)	< 0.001
Insurance type						
Health insurance	239 (74.5)	87 (82.9)	0.078	3279 (85.5)	655 (84.5)	< 0.001
Medical aid	82 (25.5)	18 (17.1)		558 (14.5)	120 (15.5)	
Comorbidity						
Myocardial infarction	17 (53.3)	48 (45.7)	0.179	1162 (30.3)	249 (32.1)	< 0.001
Congestive heart failure	144 (44.9)	48 (45.7)	0.879	819 (21.3)	198 (25.6)	< 0.001
Atrial fibrillation	59 (18.4)	20 (19.1)	0.879	365 (9.5)	97 (12.5)	< 0.001
Hypertension	290 (90.3)	93 (88.6)	0.601	2194 (57.2)	493 (63.6)	< 0.001
Peripheral vascular Disease	82 (25.6)	29 (27.6)	0.674	539 (14.1)	111 (14.3)	< 0.001
CVA or TIA	104 (32.4)	27 (25.7)	0.198	622 (16.2)	128 (16.5)	< 0.001
Diabetes mellitus	226 (70.4)	81 (77.1)	0.182	1697 (44.2)	389 (50.2)	< 0.001
Pulmonary TB	29 (9.0)	5 (4.8)	0.161	247 (6.4)	36 (4.7)	0.056
Malignancy except lung Cancer	94 (29.3)	31 (29.5)	0.963	839 (21.9)	162 (20.9)	0.001
Lung cancer	21 (6.5)	14 (13.3)	0.028	347 (9.0)	105 (13.6)	< 0.001
Death	78 (24.3)	25 (23.8)	0.919	921 (24.0)	208 (26.8)	0.244

CKD, chronic kidney disease; CVA, cerebrovascular accident; IPF, idiopathic pulmonary fibrosis; TB, tuberculosis; TIA, transient ischemic attack.

### Pirfenidone treatment in CKD patients

The use of pirfenidone was more common in the group of patients with IPF and CKD compared with the group without CKD (105 of 426 (24.7%) *vs*. 775 of 4612 (16.8%); p < 0.001). Among the 105 IPF patients with CKD who were treated with pirfenidone, 11 received RRT (10 on HD and one KT recipient). These patients had an average age of 76 years, and 70% were male. The majority of patients had comorbidities including hypertension (90%) and diabetes mellitus (100%). Four patients (40%) died.

### Effects of pirfenidone on mortality in IPF patients with or without CKD

**Figure 2:**
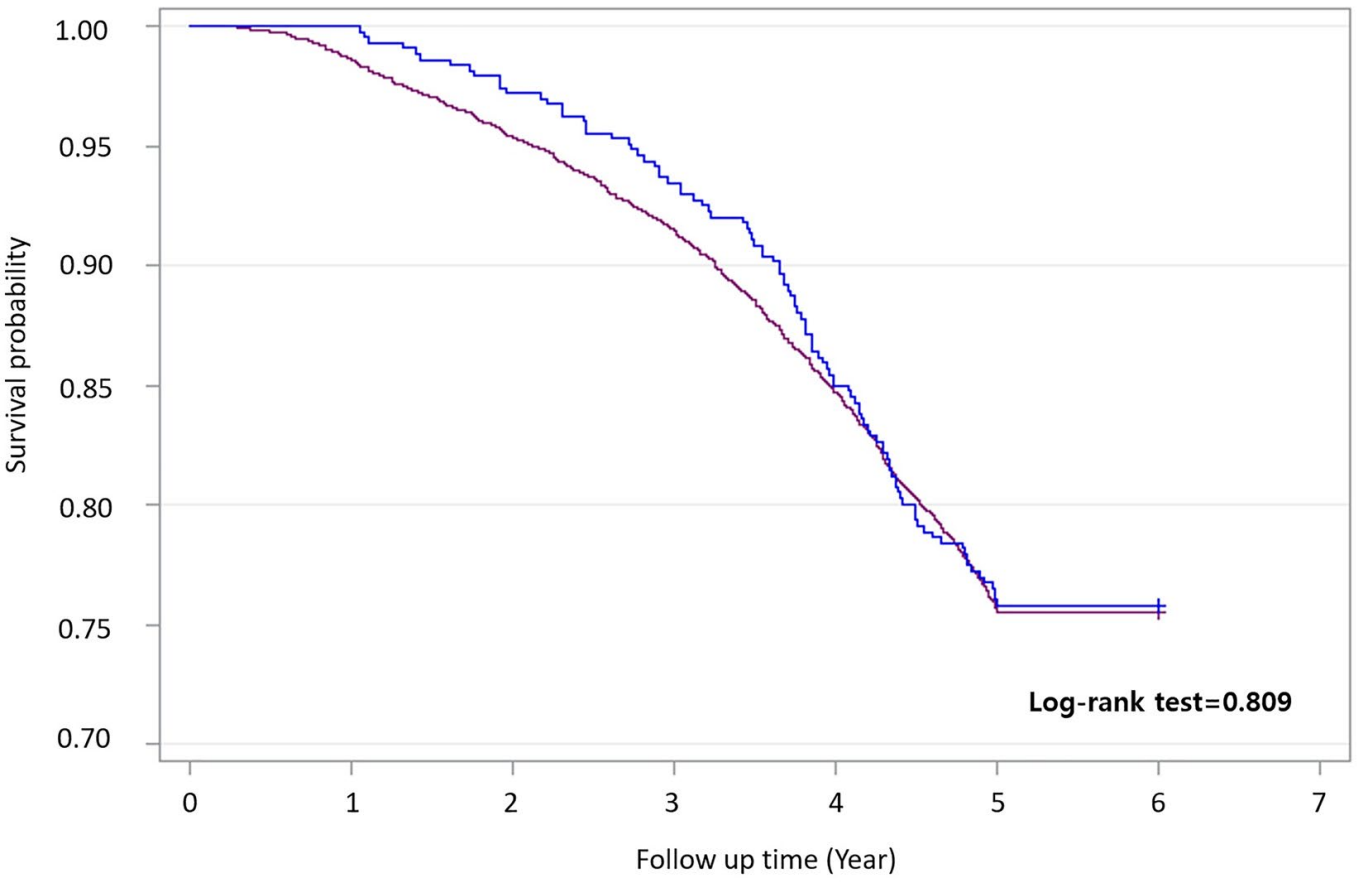
Survival curve of all-cause mortality in IPF patients with or without CKD. CKD, chronic kidney disease; IPF, idiopathic pulmonary fibrosis.

**Figure 3:**
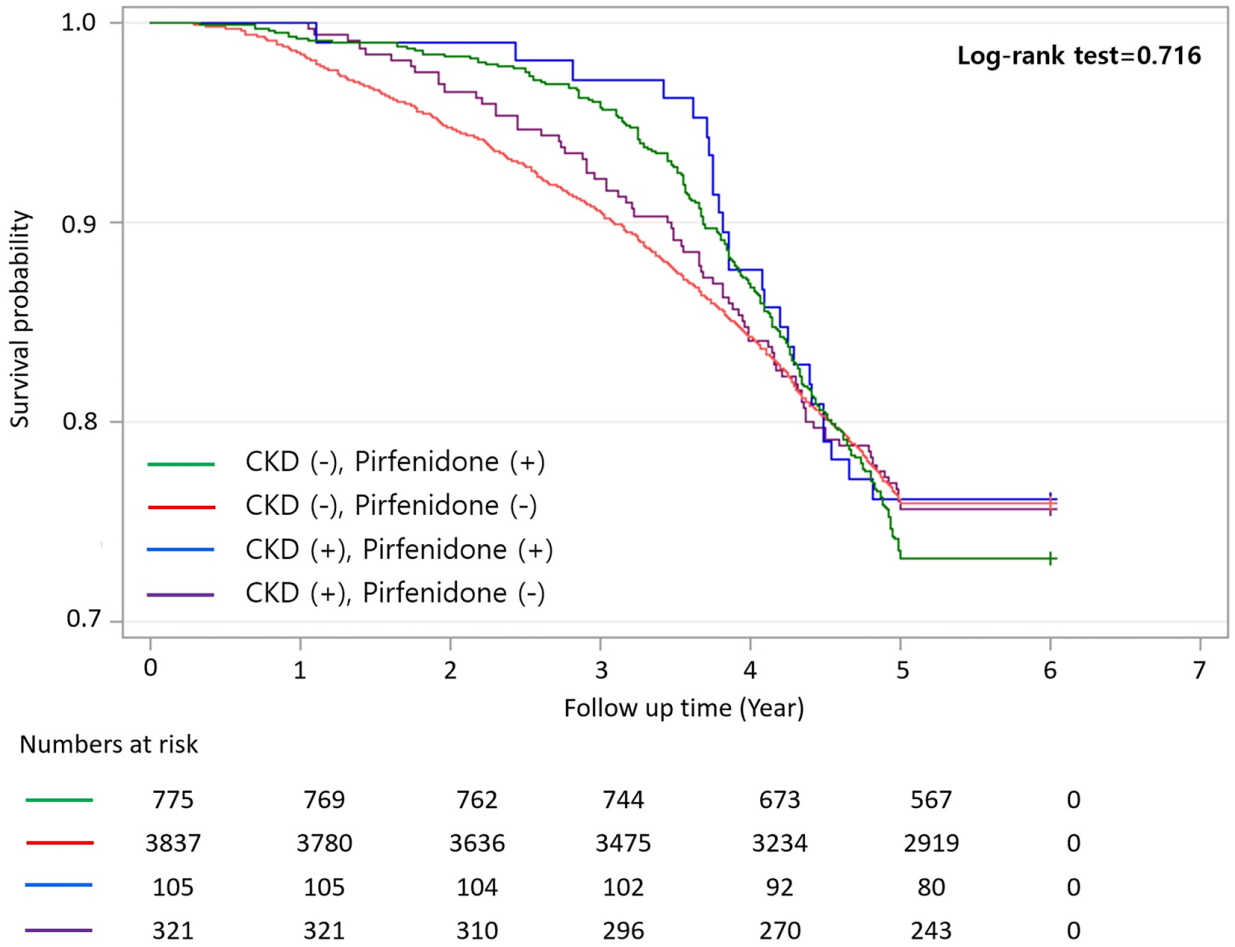
Survival curve of all-cause mortality in IPF patients with or without CKD based on pirfenidone use. CKD, chronic kidney disease; IPF, idiopathic pulmonary fibrosis.

Death occurred in 103 of 426 (24.2%) IPF patients with CKD and 1129 of 4612 (24.5%) IPF patients without CKD. Death was observed in 24.3% of IPF patients with CKD who were not prescribed pirfenidone, 23.8% of IPF patients with CKD who were prescribed pirfenidone, 24% of IPF patients without CKD who were not prescribed pirfenidone, and 26.8% of IPF patients without CKD who were prescribed pirfenidone. Significant difference was not observed in mortality between IPF patients with or without CKD (log-rank test 0.809; Fig. 2). Furthermore, significant differences were not observed among the four groups when comparing mortality based on pirfenidone treatment (log-rank test, 0.716; Fig. 3).

Comorbid CKD was not an independent risk factor for mortality in IPF patients, and pirfenidone treatment did not influence mortality in IPF patients regardless of CKD status based on Cox proportional hazards analysis (Table 2).

**Table 2:** Cox proportional hazards analysis of mortality in IPF patients.

	HR	95% CI	P value	Age, sex-Adjusted HR	95% CI	**P value**
CKD patients	0.98	(0.80, 1.19)	0.811	0.87	(0.71, 1.07)	0.179
Pirfenidone (+)	Reference			Reference		
Pirfenidone (−)	1.05	(0.67, 1.64)	0.840	0.95	(0.63, 1.55)	0.953
Non-CKD patients						
Pirfenidone (+)	1.14	(0.75, 1.73)	0.537	1.21	(0.80, 1.83)	0.375
Pirfenidone (−)	1.05	(0.70, 1.56)	0.825	1.12	(0.75, 1.67)	0.569

CI, confidence interval; CKD, chronic kidney disease; HR, hazard ratio.

## Discussion

In a large nationwide database that includes almost the entire population of Korea, most IPF patients with coexisting CKD did not receive RRT. The use of antifibrotics was more common in patients with CKD than in subjects without CKD and was only used in a few dialysis patients. Although CKD coexisting with IPF was more common in older patients and subjects with multiple comorbidities, its presence did not contribute to a worse mortality rate. In addition, the effects of pirfenidone therapy on survival did not differ between IPF patients with or without CKD.

Because curative treatment for IPF does not currently exist, non-pharmacological therapies such as oxygen supply and pulmonary rehabilitation as well as pharmacological therapies are recommended. However, treatment options with drugs remain limited to antifibrotics such as pirfenidone and nintedanib. The main goal for administering antifibrotic agents is to slow the progression of disease and to relieve symptoms^16,17^. Pirfenidone was the first agent to receive approval for treating patients with IPF and was granted priority review by the US Food and Drug Administration (FDA) in 2011^18^. However, the FDA advises caution with pirfenidone when the patient’s creatinine clearance is < 80 mL/min, while the European Medicines Agency recommends caution when creatinine clearance is between 30–50 mL/min and avoidance when creatinine clearance is < 30 mL/min^19^. Both agencies also contraindicate the use of pirfenidone in patients undergoing dialysis. The efficacy and safety of pirfenidone for the treatment of IPF have been extensively evaluated in clinical trials^20–24^, and the worsening of renal function was not listed as one of main safety concern. However, these finding do not guarantee the safety of pirfenidone in patients with CKD. This is due to a lack of safety data in CKD patients, particularly those undergoing hemodialysis, which can be attributed to the exclusion of CKD patients in most clinical trials involving pirfenidone for IPF treatment.

Pirfenidone, 5-methyl-1-phenyl-2-(1H)-pyridine, is an orally active antifibrotic agent mainly metabolized by CYP1A2, and approximately 80% of an orally administered dose of pirfenidone is excreted in urine within 24 h^25^. Reportedly, the blood concentration of pirfenidone 5-carboxy-pirfenidone metabolite excreted through the kidneys significantly increases in patients with severe renal impairment (area under the curve of mild, moderate, and severe renal impairment was 49.3, 100, and 168 mg·hr/L, respectively; all values were significantly higher than in the normal renal function group), indicating the need for caution with its use^26^. However, the role of this metabolite in the progression of renal impairment is not well understood. In addition, no relationship was found between renal insufficiency and pirfenidone level. Some retrospective studies have reported no clear effect of pirfenidone treatment on the progression of renal impairment in patients with IPF. Matsumoto et al.^27^ reviewed 93 patients who were prescribed pirfenidone for interstitial pneumonia with estimated glomerular filtration rate (eGFR) < 75 mL/min/1.73 m^2^ at the start of the treatment. There were 34 patients with CKD with eGFR < 60 mL/min/1.73 m^2^. The authors found that pirfenidone suppressed the decline in renal function for six months after initiation of treatment in patients with CKD, indicating pirfenidone as effective in protecting against the decrease of renal function in CKD patients.

Kidney fibrosis is hallmark of CKD, and preclinical studies have suggested that the overexpression of platelet-derived growth factor (PDGF) receptor-ꟗ in mesenchymal cells is associated with glomerular and interstitial fibrosis and inflammation as well as tubular atrophy^28,29^. Therefore, the use of antifibrotics as a therapeutic target for kidney fibrosis has been of interest, although the mechanism of action is not yet fully understood. Some clinical trials of pirfenidone have been conducted in patients with CKD. In a study in which the effect of pirfenidone was evaluated in 21 patients with focal segmental glomerulosclerosis^30^, the annual change in eGFR decreased from − 7.3 mL/min/1.73 m^2^ before drug use to − 5.4 mL/min/1.73 m^2^ during drug use. Three patients discontinued treatment within four months due to drug-related adverse effects, and the time to RRT initiation did not differ. In another study that included 77 patients with diabetic CKD who were randomized to receive placebo, pirfenidone 1200 mg/day, or pirfenidone 2400 mg/day for one year^31^, analysis of the 52 patients who completed treatment showed increase in mean eGFR in the pirfenidone 1200 mg/day group (+ 3.3 ± 8.5 mL/min/1.73 m^2^; p = 0.026 compared with placebo) but decreased eGFR in the placebo and pirfenidone 2400 mg/day groups (− 2.2 ± 4.8 and − 1.9 ± 6.7 mL/min/1.73 m^2^, respectively). Four of 26 patients (15%) in the placebo group and one of 25 patients (4%) in the pirfenidone 2400 mg/day group started dialysis, while no patients in the pirfenidone 1200 mg/day group required dialysis. However, nine of 25 patients (35%) in the pirfenidone 1200 mg/day group and 11 of 25 patients (44%) in the pirfenidone 2400 mg/day group discontinued drug use due to drug-related adverse effects. Although the results are limited due to a very small number of studies and patients, an ongoing phase 2 TOP-CKD trial (NCT04258397) that aims to recruit 200 participants and will be completed by December 2024, will hopefully provide additional evidence of the efficacy of pirfenidone as treatment for kidney fibrosis.

Among the 426 CKD patients in the present study, 355 (83.3%) did not require RRT or KT. In addition, the larger proportion of patients receiving pirfenidone therapy in the IPF group with CKD compared with subjects without CKD indicates that patients with relatively mild CKD were mostly included. In this study, overall mortality was not significantly different among groups irrespective of CKD and the utilization of pirfenidone treatment. However, during the initial 3-years, it appeared that patients in the pirfenidone group exhibited higher survival rates regardless of the presence of CKD, even CKD group has more comorbidities than non-CKD group. While it is plausible that pirfenidone may confer a protective effect, especially within few years of diagnosis. However, we should acknowledge the progressive nature and median survival rate of IPF. Beyond this point, approximately 3-years in our study, the influence of the underlying disease itself becomes increasingly significant, overshadowing any potential benefits associated with antifibrotic therapy. Results of this study are not informative regarding the efficacy or safety of pirfenidone use in patients with CKD, especially severe CKD patients who are currently contraindicated for antifibrotics use and might have a worse prognosis without any therapeutic interventions. Therefore, additional research on the effects of antifibrotics on renal function in IPF patients is needed before recommending the use of antifibrotics in IPF patients with CKD.

In this study, there was no difference in the effects of pirfenidone on survival between IPF patients with CKD and IPF patients without CKD. Because antifibrotic therapy is rarely used in more advanced CKD patients, results of this study highlight the need for further research on antifibrotic treatment in this population. However, the present study has several limitations. First, information regarding the severity of renal impairment based on laboratory data such as serum creatinine and GFR, as well as the severity of IPF based on pulmonary function tests and/or chest CT scan, was unattainable within the HIRA database. Consequently, we were unable to account for the impact of disease severity on mortality. Although CKD was classified based on the need for RRT or KT, an analysis on dose and/or duration of pirfenidone prescription and its effect on prognosis based on the severity of CKD could not be conducted. Most CKD patients in the study likely had mild disease because pirfenidone prescription was more frequent in patients with CKD accompanying IPF than in subjects without CKD. Additional research with a large sample size is required to encompass a diverse range of CKD patient severities. Second, mortality case was defined by no medical utilization for more than a year, then it was not feasible to analyze the precise cause of mortality. Therefore, our analysis was limited to assessing the risk of all-cause mortality. Third, this study did not include nintedanib, another antifibrotic agent, as its limited accessibility because it is not covered by health insurance in Korea. As a result, we focused on the impact of pirfenidone, the sole antifibrotic agent accessible through reimbursement. Differences in reimbursement status for antifibrotic agents between Korea and other countries may lead to varying outcomes. Fourth, patient-related factors which might influence the results as confounders, including smoking status and adherence to the pirfenidone therapy and drug related side effects which might influence adherence and difficulty in dose escalation, are not ascertainable in the claim database. Lastly, the analysis was confined to a Korean population and analyzed mortality for 6 years, rendering it challenging to generalize the findings to other racial or ethnic groups and long-term outcomes. Hence, there is a need for future multinational, long-term studies.

In conclusion, use of antifibrotics is limited in IPF patients with comorbid CKD because it is not recommended for CKD, especially in subjects who have severely compromised renal function. However, evidence is insufficient for the effects and safety concerns of antifibrotic therapy in such patients. Mortality of IPF patients did not increase with comorbid CKD and did not differ based on antifibrotic treatment. Most CKD patients were not on RRT, and the prescription rate of antifibrotics was not very high, which could weaken the observed effects of CKD in patients with IPF. Therefore, additional research on the use of antifibrotics in IPF patients with CKD is needed to expand the treatment opportunities for IPF patients with CKD.

### Supplementary Information

Supplementary Information.

## Data Availability

The data that support the findings of this study are available on reasonable request from the corresponding author.

## Abbreviations

CKD: Chronic kidney disease
CT: Computed tomography
eGFR: Estimated glomerular filtration rate
HD: Hemodialysis
HIRA: Health Insurance Review and Assessment
ICD-10: International Classification of Diseases 10th Revision
IPF: Idiopathic pulmonary fibrosis
KT: Kidney transplantation
NHIS: National Health Insurance System
PD: Peritoneal dialysis
PDGF: Platelet-derived growth factor
RRT: Renal replacement therapy

## References

[1] Raghu G (2011). An official ATS/ERS/JRS/ALAT statement: idiopathic pulmonary fibrosis: Evidence-based guidelines for diagnosis and management. Am J Respir Crit Care Med.

[2] du Bois RM (2012). An earlier and more confident diagnosis of idiopathic pulmonary fibrosis. Eur Respir Rev.

[3] Raghu G, Amatto VC, Behr J, Stowasser S (2015). Comorbidities in idiopathic pulmonary fibrosis patients: A systematic literature review. Eur Respir J.

[4] Kreuter M (2015). Treatment and outcome of lung cancer in idiopathic interstitial pneumonias. Sarcoidosis Vasc Diffuse Lung Dis.

[5] Hamada K (2007). Significance of pulmonary arterial pressure and diffusion capacity of the lung as prognosticator in patients with idiopathic pulmonary fibrosis. Chest.

[6] Lee JS (2011). Gastroesophageal reflux therapy is associated with longer survival in patients with idiopathic pulmonary fibrosis. Am J Respir Crit Care Med.

[7] Hyldgaard C, Hilberg O, Bendstrup E (2014). How does comorbidity influence survival in idiopathic pulmonary fibrosis?. Respir Med.

[8] Jovanovic DM (2022). Comorbidity burden and survival in patients with idiopathic pulmonary fibrosis: The EMPIRE registry study. Respir Res.

[9] Hill NR (2016). Global prevalence of chronic kidney disease—A systematic review and meta-analysis. PLoS One.

[10] James MT, Hemmelgarn BR, Tonelli M (2010). Early recognition and prevention of chronic kidney disease. Lancet.

[11] Foreman KJ (2018). Forecasting life expectancy, years of life lost, and all-cause and cause-specific mortality for 250 causes of death: Reference and alternative scenarios for 2016–40 for 195 countries and territories. Lancet.

[12] Tonelli M (2006). Chronic kidney disease and mortality risk: A systematic review. J Am Soc Nephrol.

[13] Navaneethan SD (2016). Obstructive and restrictive lung function measures and CKD: National Health and Nutrition Examination Survey (NHANES) 2007–2012. Am J Kidney Dis.

[14] Ikezoe K (2017). Chronic kidney disease predicts survival in patients with idiopathic pulmonary fibrosis. Respiration.

[15] Prior TS (2021). Clusters of comorbidities in idiopathic pulmonary fibrosis. Respir Med.

[16] Travis WD (2013). An official American Thoracic Society/European Respiratory Society statement: Update of the international multidisciplinary classification of the idiopathic interstitial pneumonias. Am J Respir Crit Care Med.

[17] Bradley B (2008). Interstitial lung disease guideline: The British Thoracic Society in collaboration with the Thoracic Society of Australia and New Zealand and the Irish Thoracic Society. Thorax.

[18] Food and Drug Administration. Printed labeling for Esbriet (pirfenidone) film-coated tablets. Accessed April 5, 2023. <https://www.accessdata.fda.gov/drugsatfda_docs/label/2017/208780s000lbl.pdf>

[19] European Medicines Agency. Esbriet product information. Accessed April 5, 2023. <https://www.ema.europa.eu/en/documents/product-information/esbriet-epar-product-information_en.pdf>

[20] Noble PW (2011). Pirfenidone in patients with idiopathic pulmonary fibrosis (CAPACITY): Two randomised trials. Lancet.

[21] King TE (2014). A phase 3 trial of pirfenidone in patients with idiopathic pulmonary fibrosis. N Engl J Med.

[22] Taniguchi H (2010). Pirfenidone in idiopathic pulmonary fibrosis. Eur Respir J.

[23] Ogura T (2015). All-case post-marketing surveillance of 1371 patients treated with pirfenidone for idiopathic pulmonary fibrosis. Respir Investig.

[24] Costabel U (2017). An open-label study of the long-term safety of pirfenidone in patients with idiopathic pulmonary fibrosis (RECAP). Respiration.

[25] Rubino CM, Bhavnani SM, Ambrose PG, Forrest A, Loutit JS (2009). Effect of food and antacids on the pharmacokinetics of pirfenidone in older healthy adults. Pulm Pharmacol Ther.

[26] European Medicines Agency. Pirfenidone CHMP assessment report (2010). <www.ema.europa.eu/docs/en_GB/document_library/EPAR_-_Public_assessment_report/human/002154/WC500103073.pdf>

[27] Matsumoto J, Sunohara K, Mori Y, Nagaya H, Inaba S (2021). Effects of pirfenidone on renal function in patients with interstitial pneumonia. Ren Fail.

[28] Buhl EM (2020). Dysregulated mesenchymal PDGFR-β drives kidney fibrosis. EMBO Mol Med.

[29] Kuppe C (2021). Decoding myofibroblast origins in human kidney fibrosis. Nature.

[30] Cho ME, Smith DC, Branton MH, Penzak SR, Kopp JB (2007). Pirfenidone slows renal function decline in patients with focal segmental glomerulosclerosis. Clin J Am Soc Nephrol.

[31] Sharma K (2011). Pirfenidone for diabetic nephropathy. J Am Soc Nephrol.

