# Supplementary material for: Effects of comorbid chronic kidney disease on mortality in idiopathic pulmonary fibrosis patients and influence of pirfenidone
Source: Sci Rep. 2023 Nov 7;13:19238. doi: 10.1038/s41598-023-46506-0 (PMC10630477; doi:10.1038/s41598-023-46506-0)
Supplement: Supplementary file 1 — Supplementary Information. [file 41598_2023_46506_MOESM1_ESM.docx]

**"Online Supplementary data"**

**Effects of comorbid chronic kidney disease on mortality in idiopathic pulmonary fibrosis patients and influence of pirfenidone**

**Table S1. Exclusion for connective tissue disease related and other diffuse lung parenchymal diseases**

| **Rheumatic disease or vasculitis** | ICD-10 code |
| --- | --- |
| Rheumatoid lung disease | M051 |
| Rheumatoid vasculitis | M052 |
| Rheumatoid arthritis | M053 |
| Seropositive RA | M058, M059, M060, M068, M069 |
| Polyarteritis with lung involvement | M30.1 |
| Wegener’s granulomatosis | M31.3 |
| Microscopic polyangiitis | M31.7 |
| Systemic lupus erythematosus | M329 |
| Dermatomyositis | M33 |
| Systemic sclerosis | M34 |
| Sjogren’s syndrome | M350 |
| Mixed connective tissue disease | M351 |
| **Other diffuse lung parenchymal disease** |  |
| chemicals related, pneumoconiosis, HP | J60 to J709 |
| Pulmonary alveolar proteinosis | J84.01 |
| Sarcoidosis | D86 |
| Lymphangioleiomyomatosis | J84.81 |
| pulmonary Langerhans cell histiocytosis | J84.82 |

ICD-10, International Classification of Diseases 10th Revision

**Table S2. Clinical characteristics of IPF with or without CKD**

| **Characteristics** | **IPF group** | | | **P value** |
| --- | --- | --- | --- | --- |
|  | **Total (n=5,038)** | **With CKD**  **(n = 426)** | **Without CKD (n = 4,612)** |  |
| **Patients related** |  |  |  |  |
| Age, years | 73.29 ± 9.68 | 76.77 ± 8.88 | 72.97 ± 9.69 | <0.001 |
| Sex, male (%) | 3,646 (72.4) | 355 (83.3) | 3,291 (71.4) | <0.001 |
| Insurance type |  |  |  |  |
| Health insurance | 4,260 (84.6) | 326 (76.5) | 3,934 (85.3) | <0.001 |
| Medical aid | 778 (15.4) | 100 (23.5) | 678 (14.7) |  |
| **Comorbidity** |  |  |  |  |
| Myocardial infarction | 1,630 (32.4) | 219 (51.4) | 1,411 (30.6) | <0.001 |
| Congestive heart failure | 1,209 (24.0) | 192 (45.1) | 1,017 (22.1) | <0.001 |
| Atrial fibrillation | 541 (10.7) | 79 (18.5) | 462 (10.0) | <0.001 |
| Hypertension | 3,070 (60.9) | 383 (89.9) | 2,687 (58.3) | <0.001 |
| Peripheral vascular disease | 761 (15.1) | 111 (26.1) | 650 (14.1) | <0.001 |
| CVA or TIA | 881 (17.5) | 131 (30.8) | 750 (16.3) | <0.001 |
| Diabetes mellitus | 2,393 (47.5) | 307 (72.1) | 2,086 (45.2) | <0.001 |
| Pulmonary TB | 317 (6.3) | 34 (8.0) | 283 (6.1) | 0.134 |
| Malignancy except lung cancer | 1,126 (22.4) | 125 (29.3) | 1,001 (21.7) | <0.001 |
| Lung cancer | 487 (9.7) | 35 (8.2) | 452 (9.8) | 0.290 |
| Death | 1,232 (24.5) | 103 (24.2) | 1,129 (24.5) | 0.890 |

CKD, chronic kidney disease; CVA, cerebrovascular accident; IPF, idiopathic pulmonary fibrosis; TB, tuberculosis; TIA, transient ischemic attack
